# Synthesis of Transition-Metal-Doped Nanocatalysts with Antibacterial Capabilities Using a Complementary Green Method

**DOI:** 10.3390/molecules28104182

**Published:** 2023-05-19

**Authors:** Anshul Singh, Ranjana Choudhary Ahirwar, Kavindra Borgaonkar, Neeta Gupta, Muhammad Ahsan, Jyoti Rathore, P. Das, S. Ganguly, Reena Rawat

**Affiliations:** 1 Department of Chemistry, Baba Mastnath University, Rohtak 124021, India; 2 Department of Chemistry, IPS Academy, Institute of Engineering and Science, Indore 452012, India; 3 Department of Biochemistry, Government Medical College, Latur 413512, India; 4 Department of Chemistry, Government E. Raghavendra Rao P.G. Science College, Bilaspur 495001, India; 5 Joint Doctoral School, Silesian University of Technology, 44-100 Gliwice, Poland; 6 Department of Chemistry, Government Engineer Vishwesarraiya Post Graduate College, Korba 495677, India; 7 Bar-Ilan Institute for Nanotechnology and Advanced Materials, Ramat Gan 5290002, Israel; 8 Department of Chemical Sciences, Siddhachalam Laboratory, Raipur 493221, India

**Keywords:** green synthesis, Mn-doped and undoped ZnMoO_4_ nanoparticles, antibacterial properties, photocatalytic, dye

## Abstract

A facile single-step wet chemical synthesis of a transition-metal-doped molybdate derivative was achieved via an *Ocimum tenuiflorum* extract-mediated green approach. The Synthesized nanomaterials of doped molybdate were characterized by optical and other spectroscopic techniques, which confirmed the size of nanocrystalline (~27.3 nm). The thermal stability of the nanomaterials confirmed through thermogravimetric analysis showed similarity with nanomaterials of Mn-ZnMoO_4_. Moreover, the nanoparticles displayed a non-toxic nature and showed antibactericidal activity. The impact of doping was reflected in band gap measurements; undoped ZnMoO_4_ showed relatively lower band gap in comparison to Mn-doped ZnMoO_4_. In the presence of light, ZnMoO_4_ nanomaterials a exhibited photocatalytic response to solochrome dark blue dye with a concentration of 50 ppm. OH^−^ and O_2_*^−^ radicals also destroyed the blue color of the dye within 2 min and showed potential antibactericidal activity towards both Gram-positive and Gram-negative bacteria, representing a unique application of the green-synthesized nanocatalyst.

## 1. Introduction

Zinc molybdate is one of the most useful ternary oxides in the family of transition metal elements with MoO_4_ (AMoO_4_; where A = transition metal element). ZnMoO_4_ is insoluble in water and has low toxicity; therefore, it is used as white pigment [1], as well as in various other applications such as photocatalysis [2,3,4], as phosphor for light-emitting diodes [5,6], as an electrochemical anode material for lithium batteries [7], in scintillating bolometers for double beta decay of 100 Mo [8,9], humidity sensors [10], antibacterial applications [11], biological imaging of deep tumors [12], supercapacitors [13] and as a catalyst of oxidation reactions [14,15]. MeMoO_4_ compounds (such as ZnMoO_4_) can be categorized into two groups: (i) those based on MoO_6_ octahedral units and (ii) those based on MoO_4_ tetrahedral units. The O → Mo charge transfer band occurs within UV-visible ranges and depends on Mo^6+^ ions. When molybdenum ions have tetrahedral sites, band gap occurs in the UV region. Band gap enters the visible region when more molybdenum ions are inserted into octahedral sites. As a consequence of the additional covalent character of Mo-O tetrahedral bonds over Mo-O, optical properties controlled through d-d transitions of inserted 3D metal chromophores and pigment develop via divalent cations. Thus, excited Mn^2+^ doping ions with a high quantum yield have a large band gap matrix. ZnMoO_4_ structural networks were built with [MoO_4_] tetrahedral units and used in d^10^ and [Ne] electronic configurations of Zn^2+^ [16]. Mn^2+^ ions with 3d5 configuration exhibited d-d transitions, which were forbidden by the spin rule [17]. Mn^2+^ was employed in this study due to its luminescent properties.

Tulsi (*Ocimum tenuiflorum*), which is also known as “elixir” in Ayurveda grows in southeast Asia, including in India. It is considered a quintessential apoptogenic herb. It is also widely known for its antimicrobial, antibacterial [18] and antioxidant activities [19]. Nanomaterials exhibit intriguing electronic and optical properties that render them useful in a variety of domains, including the biomedical field, photovoltaic devices, energy storage, etc. [20,21,22,23,24,25,26]. Thus, in the present study, we synthesized ZnMoO_4_ nanoparticles and Mn^2+^-doped ZnMoO_4_ from *Ocimum tenuiflorum* leaves via green synthesis. The synthesized nanoparticles were characterized by different spectroscopic techniques. In comparison, Mn-doped zinc molybdate (Eg = 4.22 eV) had higher band gap energy than zinc molybdate (Eg = 3.07 eV). FTIR spectroscopy showed the clear attachment of Mn to ZnMoO_4_. The crystalline size of both nanomaterials was 27.3 nm. According to the TGA/TDA spectra, when temperatures were high, Mn-doped zinc molybdate nanoparticles were more stable than ZnMoO_4_ nanoparticles. Photoluminescence spectra showed that Mn-doped ZnMoO_4_ was more photoluminescent in nature than ZnMoO_4_. There is a need to further explore biological and environment applications of doped and undoped zinc molybdate nanomaterials. In the presence of light and air, both Mn-doped and undoped ZnMoO_4_ nanomaterials generate OH^−^ and O_2_*^−^ free radicals, which showed photocatalytic activity that can degrade the blue color of SDB dye and penetrate the cell membrane of *E. coli* and *S. aureus* bacterial strains. Both nanomaterials inhibit bacterial strains. The presence of Mn in combination with ZnMoO_4_ enhances the photocatalytic properties and antimicrobial activity than undoped ZnMoO_4_ nanomaterial. Therefore, Mn-ZnMoO_4_ was more prominent than ZnMoO_4_ nanomaterials.

## 2. Results and Discussion

### 2.1. Characterization of Nanocomposites

**Figure 1:**
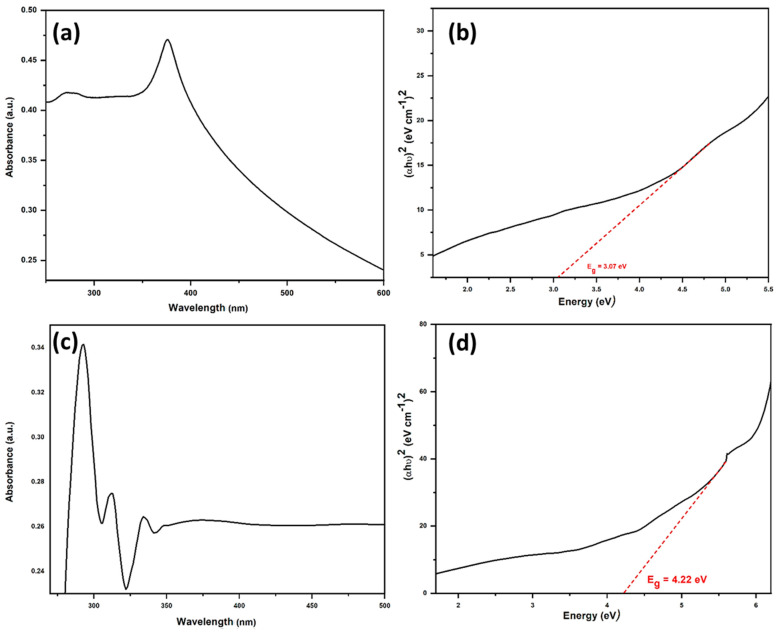
(**a**) UV−vis absorption spectrum of ZnMoO_4_. (**b**)Tauc Plot of ZnMoO_4_ derived from UV-vis absorption. (**c**) UV−vis absorption spectrum of Mn-doped ZnMoO_4_. (**d**) Tauc plot of Mn-doped ZnMoO_4_ derived from UV-vis absorption.

UV-vis spectroscopy was used to study the optical properties of synthesized zinc molybdates (Figure 1a). According to the UV spectrum, there was a sharp absorption peak at 376 nm and a broad peak at 272 nm. Typically, the optical band gap (E_g_) of nanomaterial can be estimated by the classical Tauc approach [27], which presents the relationship between the incident photoenergy (hν) and the absorption coefficient (α) near the absorption edge as follows

αhν = A_0_(hν − E_g_)^n^ (1)

It depends on the mechanism of interband transition (for example, n = 1/2 for direct transitions and n = 2 for indirect transitions). A_0_ is a constant called the band tailing parameter, and E_g_ is the intercept of the extrapolated linear when (αhν)1/n is plotted against hν. Figure 1b shows a Tauc plot of ZnMoO_4_ with a band gap value of 3.07 eV. On the other hand, Mn-doped ZnMoO_4_ nanomaterial showed an absorption peak at 293 nm and two shoulder peaks at wavelengths of 312 and 334 nm (Figure 1c). Figure 1d shows the Tauc plot of Mn-doped ZnMoO_4_ with a band gap value of 4.22 eV.

**Figure 2:**
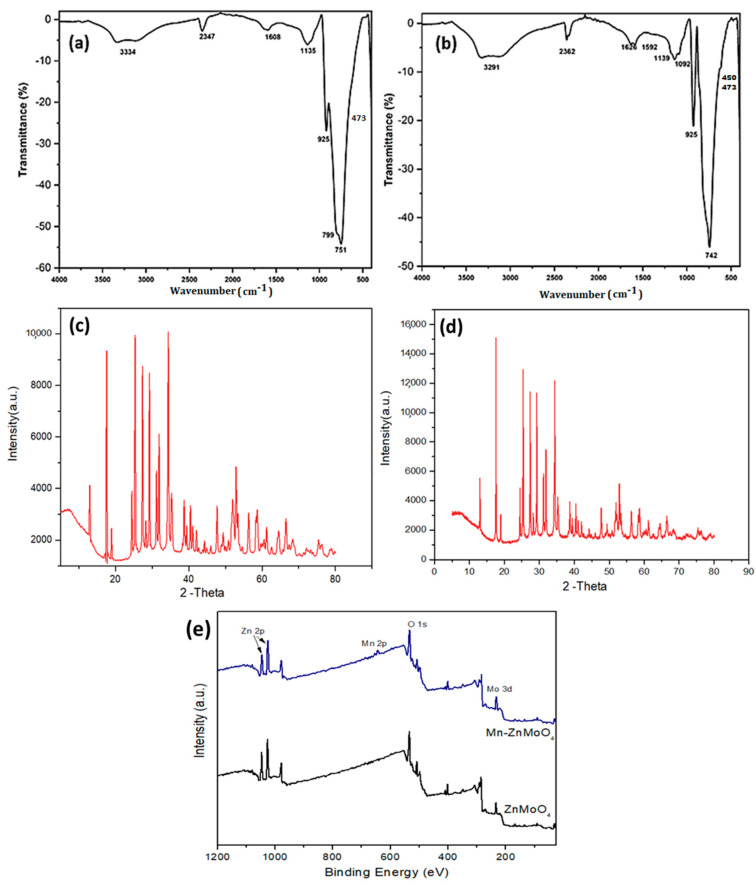
FTIR spectra of (**a**) ZnMoO_4_ and (**b**) Mn-ZnMoO_4_ nanomaterial. XRD patterns of (**c**) ZnMoO_4_ and (**d**) Mn-ZnMoO_4_ nanostructures. (**e**) Full XPS spectra of ZnMoO_4_ and Mn-ZnMoO_4_ nanomaterials.

The chemical structures of ZnMoO_4_ were identified by the FTIR spectrum. Figure 2a, corresponds to a range of 500–4000 cm^−1^. Several absorption bands were observed, such as infrared bands at 3334 and 1608 cm^−1^ that correspond to the OH stretching vibration and bending vibration of water molecules (H-O-H) [28]. Bands at 1135, 925, 799 and 751 cm^−1^ are due to [MoO_y_]^n−^, and that at 473 cm^−1^ is attributed to Zn-O in zinc molybdates [29,30,31]. The bands at 2347 are attributed to organic contamination during sample preparation. Figure 2b shows the FTIR spectrum of Mn-doped ZnMoO_4_, which resembles ZnMoO_4_. However, the identical peaks at 473 and 450 cm^−1^ are attributed to Zn-O and Mn-Zn in Mn-doped ZnMoO_4_ nanomaterial.

The X-ray diffraction (XRD) method was used to analyze the resultant complexes. According to Figure 2c, the synthesized zinc molybdate nanomaterials were crystalline in nature [32,33]. The identical XRD peaks at 2θ values of 12.9, 17.5, 25.4, 27.3, 29.3, 31.9, 34.3, 40.4, 51.9 and 52.8 belong to planes (001), (101), (112), (004), (114), (211), (200), (312) and (224) and (43), respectively. The crystalline size of zinc molybdate was 24.9 nm at 2θ = 27.3°. According to Figure 2d, the XRD spectra of Mn-doped zinc molybdate resemble those of zinc molybdate. The XRD peaks at 27.3, 29.3 and 31.7 indicate the presence of zinc molybdate nanomaterials in Mn-doped zinc molybdate. However, interlayer spacing (d) was 0.247 nm and 0.372 nm for ZnMoO_4_ and Mn-doped ZnMoO_4_, respectively.

Figure 2e shows the XPS spectra curves of ZnMoO_4_ and Mn-ZnMoO_4_. The spectra for both ZnMoO_4_ and Mn-ZnMoO_4_ display peaks of Zn2p, Mo3d and O1s [34], whereas a peak of Mn was detected in the Mn-ZnMoO_4_ sample but was absent in the ZnMoO_4_ spectrum [35].

**Figure 3:**
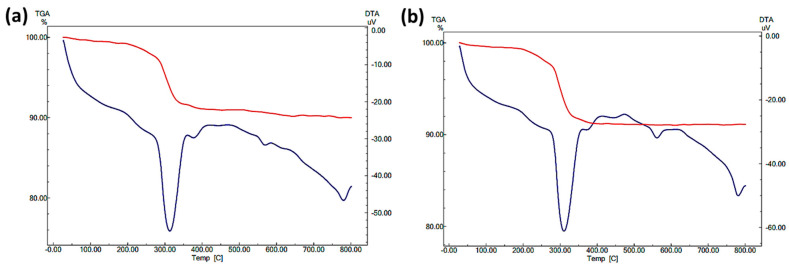
TGA and DTA analysis of (**a**) ZnMoO_4_ nanomaterials and (**b**) Mn-doped ZnMoO_4_ nanomaterials. TGA is represented by the red line, and DTA is represented but the dark blue line.

The thermal stability of green synthesized nanomaterials of zinc molybdate (ZnMoO_4_) and Mn-doped zinc molybdate (Mn-ZnMoO_4_) were characterized by TGA and DTA analysis. According to Figure 3a,b, the TGA spectra of ZnMoO_4_ and Mn-doped ZnMoO_4_ exhibited weight loss in four steps. The total weight loss was found to be around 10%.

The first weight loss was observed at temperatures greater than 150 °C due to the removal of physically adsorbed hydrated water from the surface. The second weight loss was observed at temperatures above 250 °C due to the removal of lattice water. The third weight loss was observed at temperatures above 350 °C due to hydroxide decomposition and partial removal of residues and evaporation of various gases, such as NO_2_, CO_2_, NH_3_, etc. The fourth weight loss was observed at temperatures of 520 °C due to phase transformation [36]. These results demonstrate that zinc molybdate and Mn-doped zinc molybdate nanoparticles were more thermally stable at higher temperatures.

In the DTA spectra, we observed a shift in transition temperature because of the fast heating rate. The thermal differential endothermic signal was observed to be distributed over a wide temperature range (260 °C). In the slow cooling process, we observed exothermic peaks because of crystallization (768 °C) and phase transition.

**Figure 4:**
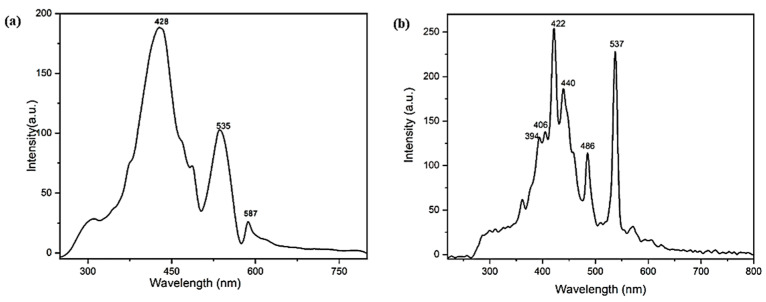
Photoluminescence spectra of (**a**) ZnMoO_4_ and (**b**) Mn-doped ZnMoO_4_.

Figure 4a,b show the emission spectra of the ZnMoO_4_ and Mn-doped ZnMoO_4_, which were excited at λ = 200 nm at room temperature [37]. The recombination of ē + h pairs in complex [MoO_4_] caused the emission bands of ZnMoO_4_ [38]. During the excitation process, some electrons located near the valence band (VB) in the 2p orbitals of the O absorbed energy (hv) and were promoted to unoccupied levels near the conduction band (CB) in Mo 4d orbitals. Electrons participated in the emission processes, which involved recombination phenomena in centers located in the band gap, and increases the recombination rate and an intensity of photoluminescence [39].

According to the ZnMoO_4_ (Figure 4a) emission spectrum, at 200 nm excitation, a sharp peak was emitted at 422 nm, which belongs to Mo (4d) → O(2P) transition. It is also evident that another emission peak was emitted at 535 nm, which belongs to ^5^D_3_–^7^F_6_ transition, and at 587 nm, which belongs to ^5^D_4_–^7^F_4_. According to the emission spectrum of Mn-doped ZnMoO_4,_ at 200 nm excitation, sharp emission peaks were observed at 422, 440, 486 and 537 nm (Figure 4b).

### 2.2. Antibacterial Activities of ZnMoO_4_ and Mn-Doped ZnMoO_4_

#### 2.2.1. Bacterial Species Collection

Overall, two *E. coli* and *S. aureus* strains were analyzed to show the antibacterial activity of both doped and undoped nanomaterials. The strains were previously isolated from patients with urinary tract infections and sewage water.

#### 2.2.2. Antibacterial Effect

The antibacterial activities of ZnMoO_4_ and Mn-doped ZnMoO_4_ both prevented the further growth of the two bacterial strains, i.e., *E. coli* and *S. aureus*, by inhibiting protein synthesis [40].

**Figure 5:**
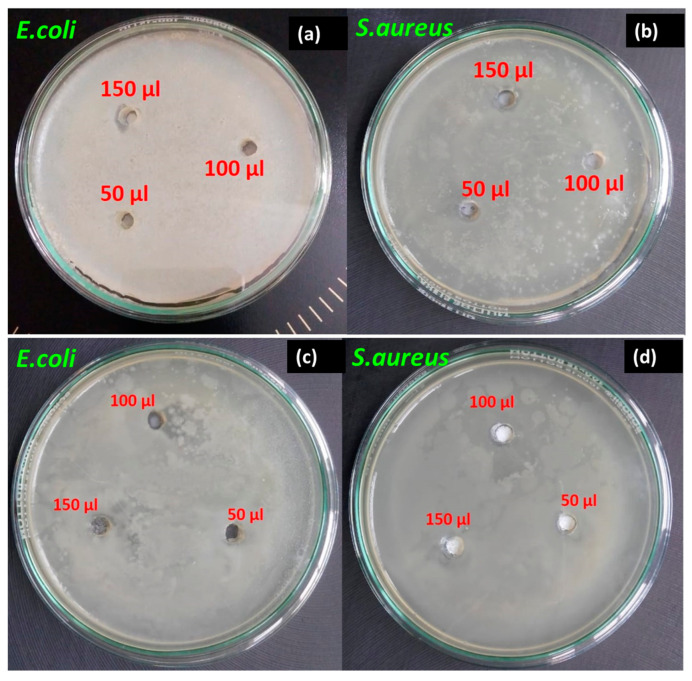
Antibacterial activity of (**a**,**b**) ZnMoO_4_ and (**c**,**d**) Mn-doped ZnMoO_4_ nanomaterials against *E. coli* and *S. aureus*.

As shown in Figure 5 different ZOIs (zones of inhibition) for antibacterial activity were obtained for ZnMoO_4_ and Mn-doped ZnMoO_4_ with different concentrations (50, 100 and 150 µg/mL) in methanol (Table 1).

It was clear that ZnMoO_4_ produced a minimum ZOI for *E. coli* (Figure 5a). However, in the case of *S. aureus*, there was a good ZOI and a better response (Figure 5b). The clear area around the sample indicates complete inhibition. Comparatively, Mn-doped ZnMoO_4_ (Figure 5c,d) showed a good ZOI and a better response for the bacterial response to both (a) *E. coli* and (b) *S. aureus*. Therefore, antibacterial activity was higher for Mn-doped ZnMoO_4_ than ZnMoO_4_ against both bacterial strains.

### 2.3. Photocatalytic Activity

Photocatalytic experiments were conducted on SDB dye using ZnMoO_4_ and Mn-doped ZnMoO_4_.

**Figure 6:**
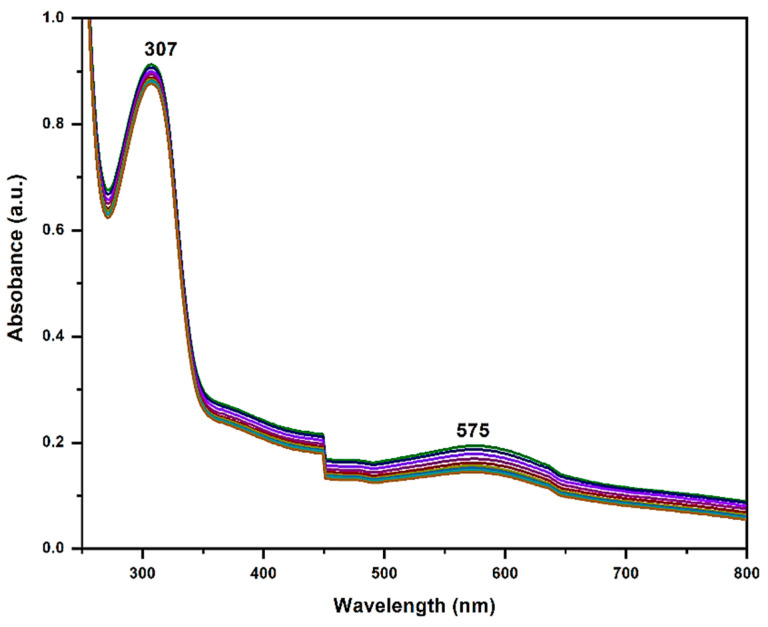
Photocatalysis reaction of SDB dye in the presence of light and air.

Figure 6 shows that the photocatalytic reaction of 50 ppm SDB dye in the presence of light after 360 min resulted in slight changes. After the addition of 40 mg ZnMoO_4_ or Mn-doped ZnMoO_4_, a drastic degradation was observed in the presence of light and air during the process of photocatalysis. The photocatalytic activity of the molybdates was due to transfer of charges from the valence band orbital of O2p to the empty conduction band orbital of Mo 4d (2p 4d) (49). Here, we describe the photocatalytic activity of ZnMoO_4_ on solochrome dark blue in by process of photodegradation.

**Figure 7:**
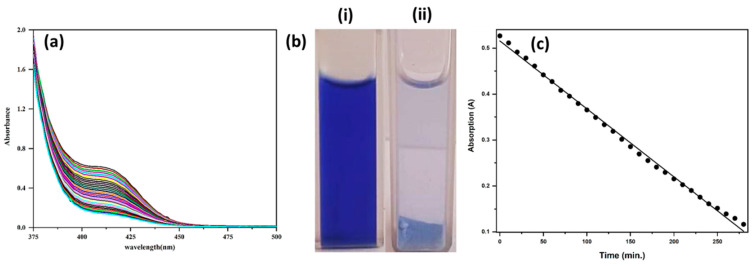
(**a**) Photocatalytic SDB degradation of ZnMoO_4_ under visible light and light at a 420 nm wavelength. (**b**) With the naked eye: (i) aq. solution of 50 ppm SDB (blank); (ii) after 120 min, the blue color of the solution degraded with 40.0 mg undoped ZnMoO_4_. (**c**) At 420 nm wavelength after adding ZnMoO_4_ in solution with increased time, there was decrease in the absorption in the presence of light and air.

In the presence of light and air, 40 mg ZnMoO_4_ was added to 10.0 mL aq. solution of 50 ppm SDB (pH = 6). With a constant time (10 min) interval, ZnMoO_4_ degraded the SDB (Figure 7a). After 120 min, the blue solution became colorless (Figure 7b). With increased time, a decrease in the absorption of blue color was observed at a constant time interval and a 420 nm wavelength (Figure 7c).

**Figure 8:**
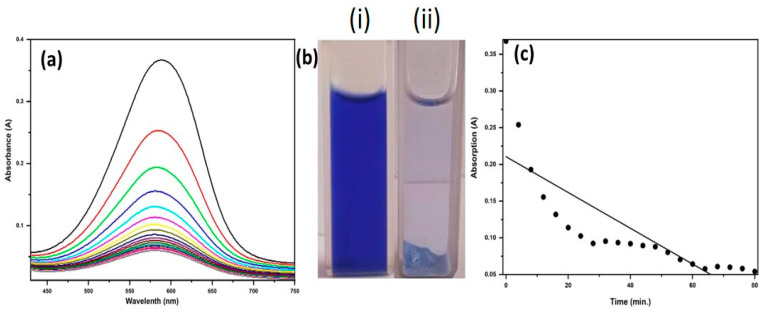
(**a**) Photocatalytic SDB degradation of Mn-doped ZnMoO_4_ under visible light and light at a 588 nm wavelength. (**b**) With the naked eye: (i) aq. solution of 50 ppm SDB (blank); (ii) after 80 min, the blue color of solution degraded with 40.0 mg undoped ZnMoO_4_. (**c**) At 588 nm wavelength, after the addition of Mn-doped ZnMoO_4_ in solution, there was decrease in absorption with an increase in time in the presence of light and air.

For comparison, 40.0 mg Mn-doped ZnMoO_4_ was added to 10.0 mL aq. Solution with a concentration of 50 ppm SDB (pH = 6) in the presence of light and air with a constant time (10 min) interval. Mn-doped ZnMoO_4_ degraded the dark blue color of the solution at a wavelength of 588 nm (Figure 8a). After 80 min, the blue solution turned colorless (Figure 8b). With increased time, a decrease in the absorption of blue color was observed when the time interval was held constant with a wavelength of 588 nm (Figure 8c).

According to absorption spectra of Mn, ZnMoO_4_ nanomaterial showed one absorption peak at 293 nm and two shoulder peaks at wavelengths of 312 and 334 nm, with a band gap value of 4.22 eV. In the case of undoped ZnMoO_4_, there was one sharp peak and one small, broad peak at 376 and 272 cm^−1^ wavelengths, respectively, with a band gap value of 3.07 eV. FTIR spectra showed identical peaks at 473 and 450 nm, which were attributed to Zn-O and Mn-Zn, respectively, for Mn-doped ZnMoO_4_ nanomaterial. At high temperatures, Mn-doped zinc molybdate nanoparticles were more stable than undoped ZnMoO_4_. According to the photoluminescence spectra, Mn-doped ZnMoO_4_ was more photoluminescent than ZnMoO_4_. Undoped ZnMoO_4_ had a characteristic band, and a sharp peak was observed at 422 nm, which belonged to the Mo (4d) → O(2P) transition. It was also evident that there were emission peaks at 535 nm and 587 nm, which belonged to ^5^D_3_–^7^F_6_ and ^5^D_4_–^7^F_4_ transitions, respectively. However, in the case of Mn-doped ZnMoO_4_, at an excitation of 200 nm, sharp emission peaks were observed at 422, 440, 486 and 537 nm.

**Figure 9:**
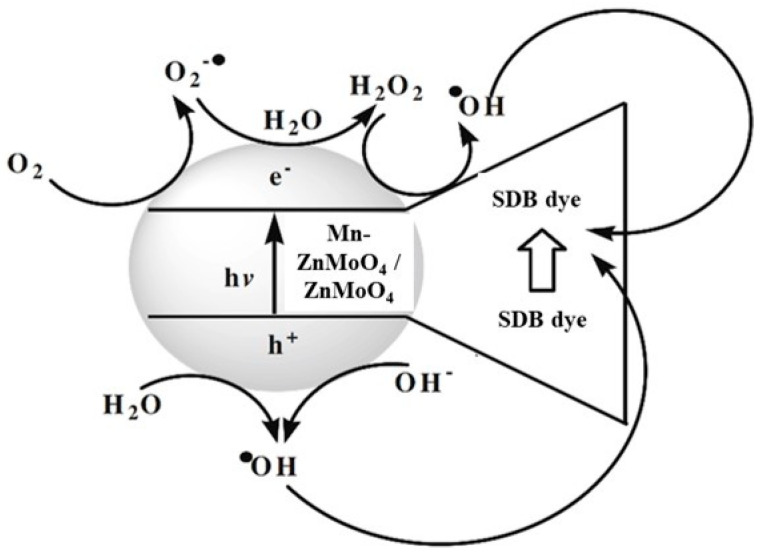
Mechanism of the photocatalytic degradation process of solochrome dark blue (SDB) dye with ZnMoO_4_/Mn-ZnMoO_4_ nanomaterials.

In ZnMoO_4_, absorbed photons were equal to or greater than its band gap energy, and electrons were excited from the VB band to the conduction band (CB), leaving a “hole” in the VB of ZnMoO_4_. These pairs of electron holes normally recombine rapidly; therefore, the photocatalytic activity of the material decreases. The photogenerated e^−^ and h^+^ reacted with H_2_O, O_2_ and the organic substrate adsorbed on photocatalytic surface for generation of reactive species such as OH and O_2_^−^. The oxidative action of OH and O_2_^−^ decomposed organic compounds into degradation products [41]. These radicals may be involved in processes of organic compound mineralization (Figure 9). Since SDB is cationic in nature, it is readily absorbed on the surfaces of catalytic ZnMoO_4_ and Mn-ZnMoO_4_ at alkaline pH, which accelerates the process of photodegradation [42,43].

The probable mechanism of photocatalytic degradation of solochrome dark blue can be summarized as follows:

ZnMoO_4_ + hν → ZnMoO_4_^−^ + e^−^ + h^+^

h^+^ + H_2_O → OH^−^

e^−^ + O_2_ → O_2_*^−^

OH^−^ + O_2_*^−^ + SDB → degradation in solution

In the presence of light and air, ZnMoO_4_ (40.0 mg) was activated to form OH^−^ and O_2_*^−^ radicals. After 120 min, the blue solution of the monochromatic dark blue dye deteriorated, and the solution became colorless. On the other hand, a photocatalytic process was developed for Mn-ZnMoO_4_ nanomaterials that follow the same photocatalytic process and also generate OH^−^ and O_2_*^−^ radicals. Due to the presence of Mn, the blue color of the SDB dye was degraded by the Mn-ZnMoO_4_ nanomaterials after 80 min, resulting in a colorless dye, which makes Mn-doped ZnMoO_4_ more important than ZnMoO_4_ in the photocatalytic process.

Free-radical °OH and O_2_° produced by non-toxic Mn-doped ZnMoO_4_ and undoped ZnMoO_4_ nanomaterials entered the cell membrane of bacterial cells and inhibited cell growth of Escherichia coli and Staphylococcus aureus species. In contrast, the difference between Mn-ZnMoO_4_ nanomaterials inhibited bacterial cell growth more than ZnMoO_4_ (undoped) (Table 1) because more free radicals were produced, which easily penetrated the cell membrane and inhibited cell growth.

## 3. Materials and Methods

### 3.1. Chemicals and Reagents

The raw materials used in the synthesis procedure were zinc sulphate (ZnSO_4_·7H_2_O; Merck 99.8%), sodium molybdate (Na_2_MoO_4_·2H_2_O; Merck 99.8%), manganese sulphate (MnSO_4_; Merck 99.5%), ethylene glycol (Merck), urea (Hi-media), *E. coli* and *S. aureus* pure culture, natural agar media (Hi-Media) and triple-distilled water.

### 3.2. Instrumentation

In the absorbance mode, UV-visible spectra were acquired using a UV-1900i double-beam spectrophotometer. Samples were dispersed in ethanol to determine absorbance. Photoluminescence measurements of powder were performed using 266 nm radiation from an Nd: YAG laser and detected by a CCD (charge coupled device) detector (model: QE 65000, Ocean Optics, Orlando, FL, USA) attached to the fiber sample, which was analyzed using an Advanced D8 Bruker X-ray diffractometer (XRD) with Ni-filtered Cu-K (1.5405) (2–10–80°; step size, 0.02°). The vibration spectra were recorded using an Avtar 370 Thermo Nicolet Fourier transform infrared (FT-IR) spectrophotometer equipped with a DTGS detector with a set resolution of 4 cm^−1^, and the samples were prepared as KBr discs for this study.

### 3.3. Extraction of Ocimum Tenuiflorum Leaves

Leaves of Ocimum Tenuiflorum were collected in January 2022 from a natural ecosystem at India. Plant leaves were washed with double-distilled water to remove impurities and dried at room temperature (25 °C). Subsequently, the washed plant leaves (100 g) were boiled with distilled water (110 mL) at 40–50 °C. The whole extract solution was filtered using Whatman filter paper No. 42 and stored at 4–5 °C. Then, the extracted filtrate was used in the synthesis of zinc molybdate nanomaterials.

### 3.4. Synthesis of Sodium Zinc Molybdate

**Scheme 1:**
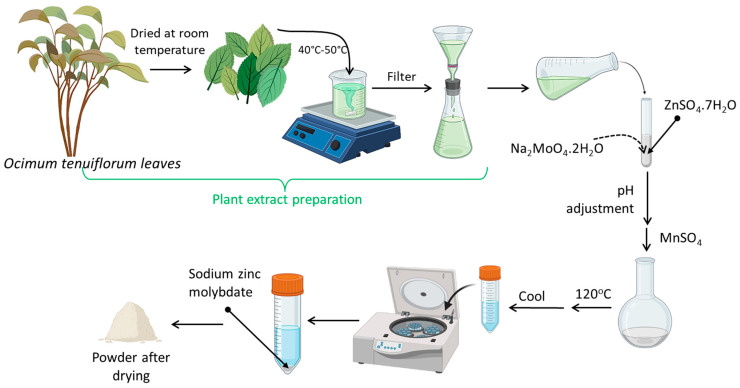
Graphical illustration of green synthesis procedure for Mn-doped zinc molybdate.

The total synthesis process is graphically illustrated in Scheme 1. Green synthesis of zinc molybdate and Mn-doped zinc molybdate NPs was carried out using crude plant extract (100 mL) heated (40–60 °C) on a magnetic stirrer with continuous stirring using a magnetic bar; then, the temperature was maintained at 50 °C. A solution of ZnSO_4_·7H_2_O and ethylene glycol was added to aforementioned solution. The pH of the solution was adjusted to 9 with urea. The entire solution was heated for an hour at 80 °C with stirring. White precipitate was developed after the addition of 2 M solution of Na_2_MoO_4_·2H_2_O. The solution was then transferred to a round-bottom flask and heated up to 120 °C with continuous stirring. The white precipitate was allowed to settle; then, the mixture was filtered to separate the precipitate from the solution. The precipitate was washed with deionized water, methanol and acetone. The resultant material was dried in an oven at 60 °C for 2 h.

### 3.5. Synthesis of Mn-Doped Sodium Zinc Molybdate

In a beaker, solutions of 1 M ZnSO_4_·7H_2_O and ethylene glycol (10 mL) were mixed together, followed by the addition of 2 M Na_2_MoO_4_·2H_2_O. After the addition of urea, the pH of the solution was adjusted to 9, followed by heating to 80 °C for one hour. The solution was stirred well with 1 gm of MnSO_4_. This solution was transferred to a round-bottom flask and heated up to 120 °C with continuous stirring. The solution was allowed to stand until white precipitate settled, which was subsequently and filtered from the solution. The precipitate was washed with deionized water, methanol and acetone. The resultant material was dried in an oven at 60 °C for 2 h.

### 3.6. Antibacterial Activity

Antibacterial activity was determined in a well diffusion assay on nutrient agar medium (NAM). The medium was cast into a Petri dish under sterile conditions and kept up to 1 h for solidification. Then, fresh overnight cultured *E. coli* and *S. aureus* (100 µg/mL) were spread onto a solidified nutrient agar Petri dish. The dishes were kept up to 15–20 min for complete absorption of bacterial cultures. Wells were prepared by gel puncture (7–8 mm). Then, ZnMoO_4_ and Mn-ZnMoO_4_ nanomaterials with different concentrations (50, 100 and 150 µg/mL) were introduced into these wells. The Petri dishes with medium were kept at room temperature for 30 min to allow for the diffusion of the extracts. Then, incubation was carried out at 37 °C for 24 h to allow for maximum growth of the microorganisms. Both nanomaterials inhibited the growth of microorganisms, displaying a clear zone of inhibition (ZOI) around the well after incubation.

### 3.7. Dye Remediation

Solochrome dark blue dye (SDB) adsorption experiments were conducted under batch conditions using green-synthesized ZnMoO_4_ and Mn-ZnMoO_4_ nanomaterials. A standard solochrome dark blue dye (SDB) stock solution was diluted with deionized water in order to obtain different concentrations. The obtained solochrome dark blue dye (SDB) solutions were kept in a flask with a fixed volume (10 mL of 5 ppm), and ZnMoO_4_ and Mn-ZnMoO_4_ nanomaterials were added. The flask was placed in a sonicator for 120 min at a pH of 6 at room temperature. After a certain time, the upper layer of liquid was analyzed by a UV-vis spectrophotometer (UV-Visible 1900i, Shimadzu, Japan) at a wavelength of 600 nm. The ZnMoO_4_ and Mn-ZnMoO_4_ were removed with the help of centrifugation after the completion of experiment. The removal (R, %) was calculated according to Equation (2) as follows:

(2) $$R\left(\%\right)=\frac{Co-Ce}{Co}\times 100$$

where *Co* and *Ce* are the initial and equilibrium concentrations of SDB (mg L^−1^), respectively.

## 4. Conclusions

In this paper, we described the green synthesis of ZnMoO_4_ and Mn-ZnMoO_4_ nanomaterials from *Ocimum tenuiflorum* (Tulsi), as well as their characterization by UV-vis absorption spectroscopy, FTIR spectroscopy, X-ray diffraction (X-ray diffraction) and luminance spectra (PL). The band gaps of ZnMoO_4_ and Mn-ZnMoO_4_ were 3.07 and 4.22 eV, respectively. Both doped and undoped nanomaterials have potential for biological and environmental applications. Both nanomaterials exhibited antibacterial activity and photocatalytic properties.

In contrast, Mn-ZnMoO_4_ nanomaterials responded better to two different bacterial species: (a) *Escherichia coli* and (b) *Staphylococcus aureus*. ZnMoO_4_ showed a positive response only against Staphylococcus aureus. Due to the formation of reactive oxidative species such as OH and O_2_ radicals, both nanomaterials were found to have photocatalytic properties, as demonstrated by solochrome dark blue dye (SDB) in the process of photodegradation. In the presence of light and air, after 80 min, Mn-doped ZnMoO_4_ (Mn-ZnMoO_4_) resulted in the decolorization of the blue dye, whereas ZnMnO_4_ took 120 min to induce decolorization.

## Figures and Tables

**Table 1:** Zones of inhibition (ZOIs) of antimicrobial activity for ZnMoO_4_ and Mn-doped ZnMoO_4_ at different concentrations in methanol solvent (50, 100 and 150 µg/mL).

Bacterial Species	Concentration of Mn-Doped ZnMoO_4_		
	50 µL	100 µL	150 µL
*E. coli*	3.0 mm	1.0 mm	1.5 mm
*S. aureus*	1.4 mm	6.0 mm	8.0 mm

## Data Availability

Not applicable.
